# MULTIDISCIPLINARY PERSPECTIVES ON LATER-LIFE DEPRESSION: THE ROLE OF BRAIN HEALTH

**DOI:** 10.1093/geroni/igac059.1085

**Published:** 2022-12-20

**Authors:** Ann Steffen, Jennifer Moye

## Abstract

Bi-directional associations between depression and cognitive functioning are magnified among aging individuals, challenging behavioral health providers who treat older adults experiencing clinical and subsyndromal depression. This symposium contributes to the science and practice of assessing and treating later-life depression while also attending to issues in professional training. The first paper presents pre-treatment data from the multi-site Optimum Study of older adults experiencing treatment-resistant depression (n = 529). The relevance of positive psychological constructs is supported with analyses showing important relationships among cognitive functioning, social participation, and psychological well-being. The second paper describes the development of an updated measure to assess behavioral health providers’ knowledge of later life depression, including brain health concerns. Psychometric data for the measure were generated from a random pool of licensed social workers (N=900) who were mailed the survey packet. The third presentation features an experimental study demonstrating that foundational information about aging, including debunking misconceptions about cognitive aging, influenced continuing education preferences of generalist Licensed Professional Counselors (LPCs). Among the randomly generated pool of LPCs (N = 120), participants who received aging-specific information were more likely to later choose an aging-specific continuing education option. The fourth paper highlights recommendations for mental health practitioners working in primary care and general medical settings with older adults who have co-existing depressive symptoms and cognitive concerns. The fifth and final presentation describes cognitive behavioral clinical tools to address brain health concerns in the context of later-life depression, using the new Brain Health module of a published client workbook.

